# Phylogenetic diversity and community structure of *Planctomycetota* from plant biomass-rich environments

**DOI:** 10.3389/fmicb.2025.1579219

**Published:** 2025-05-29

**Authors:** Dominika Klimek, Olga Maria Lage, Magdalena Calusinska

**Affiliations:** ^1^Environmental and Industrial Biotechnology, Luxembourg Institute of Science and Technology (LIST), Esch-sur-Alzette, Luxembourg; ^2^Faculty of Science, Technology and Medicine (FTSM), University of Luxembourg, Esch-sur-Alzette, Luxembourg; ^3^Department of Biology, Faculty of Sciences, University of Porto, Porto, Portugal; ^4^Interdisciplinary Centre of Marine and Environmental Research (CIIMAR/CIMAR), Porto, Portugal

**Keywords:** *Planctomycetota*, planctomycetes diversity, taxonomic profiling, 16S rRNA gene sequence analysis, microbial community structure, biomass-rich environments, anaerobic digester, termite gut

## Abstract

Biomass-rich environments host diverse microbial communities that contribute to the degradation and recycling of organic matter. Understanding the community structure within these habitats is essential for elucidating the ecological roles and metabolic capacities of specific microbial groups. Here, we conducted an analysis of biomass-rich environments including diverse soil types, sediments, anaerobic digesters, termite guts, termite nests and other decaying biomasses, to explore the phylogenetic diversity and community structure of the *Planctomycetota* phylum, using short-read 16S rRNA gene amplicon sequencing. All sampled environments showed presence of *Planctomycetota*, with relative abundance ranging from nearly absent in animal manure to approximately 10% in soils. Across all samples, virtually 1,900 operational taxonomic units (OTUs) were identified, classified into diverse classes within *Planctomycetota*. Planctomycetotal phylogenetic diversity was highest in soils and sediments, while termite guts, exhibiting the lowest phylogenetic diversity, were dominated by a few core OTUs shared across different termite species. Notably, a single OTU, closely matching the 16S rRNA gene sequence of the *Singulisphaera* genus, was detected in all environments, though with relative abundance ranging from only a few reads to over 6% of the planctomycetotal community. Four environments such as soil, sediment, termite nest and decaying biomasses showed similar community structure with predominant genera such as *Tepidisphaera, Telmatocola*, and distantly related to *Thermogutta*, and *Anatilimnicola*. However, among these environments, weighted UniFrac analysis revealed that planctomycetotal communities in termite nests exhibited greater phylogenetic relatedness. Termite gut communities were the most divergent, followed by those in anaerobic digesters, where OTUs assigned to *Anaerobaca* and *Anaerohalosphaera* were the most abundant. Termite gut and phytoplankton bloom samples were dominated by OTUs affiliated with *Pirellulales*, suggesting their host-specific associations. Animal manure showed the presence of *Planctomycetota*, with 25% of detected OTUs not recognized by the SILVA database, possibly representing a novel, host-specific lineage distantly related to the *Pirellulales* order.

## Introduction

Biomass can be defined as organic matter from living organisms like plants, animals or microorganisms that can be processed (Bonechi et al., 2017). Plant biomass, mainly lignocellulose, represents a vital form of renewable energy, and it has been increasingly used as a feedstock for a wide range of energy products (Bajpai, 2020). Carbohydrates make up the largest fraction of plant biomass, with cellulose and hemicellulose being the most abundant components. Cellulose microfibrils, embedded in a matrix of hemicellulose and lignin, form a highly resistant and intertwined structure that poses challenges for lignocellulose degradation and conversion processes (Chen, 2014). The particular components of plant biomass drive the structure and dynamics of microbial communities in diverse environments. Thus, studying the bacteria in environments with high organic loads, such as those rich in primary and secondary plant biomasses, is crucial for understanding their roles in biomass degradation (Lillington et al., 2020; Wang et al., 2016; Weimer, 2022). This is specifically insightful, as biomass-rich systems impose unique selective pressures that may drive bacteria along distinct evolutionary pathways, thereby fostering the emergence of diverse and potentially novel bacterial lineages (Martin and Sousa, 2016). *Bacteroidota* serves as an excellent example of a widespread phylum in which only certain lineages are known biomass degraders, often targeting different fractions of organic matter (Fernández-Gómez et al., 2013). For instance, marine members of *Bacteroidota*, such as the *Flavobacteriia* class, specialize in degrading high-molecular-weight organic matter from algal blooms, while *Bacteroides* genus is adept at degrading complex dietary polysaccharides including lignocellulose (Briliūtė et al., 2019; Kappelmann et al., 2019).

Microbial communities are pivotal in decomposing biomass and cycling nutrients across varied environments, each with distinct ecological roles. In soil, microbes break down complex plant materials, releasing essential nutrients, enhancing soil structure, and finally supporting plant growth (Žifčáková et al., 2017). Composting is an aerobic process which relies on microbial activity to decompose organic matter, producing a stable, nutrient-rich humus that enhances soil fertility and structure (Partanen et al., 2010). Built environments, such as landfills and anaerobic digestion (AD) reactors, host diverse microbial communities that play essential roles in organic matter decomposition, fermentation and methane production (Nguyen et al., 2019; Wang et al., 2017). In the animal gut, particularly in herbivores, specialized microbial community ferments fibrous biomass, producing short-chain fatty acids that supply energy to the host (Lee and Hase, 2014). In termites, this mutualistic relationship is taken to an extraordinary level – their gut functions as one of the smallest and most efficient bioreactors on the planet, housing a highly specialized microbiota capable of breaking down lignocellulosic biomass, particularly wood, which few other animals can digest (Goux et al., 2023). The lignocellulose-degrading microorganisms are also considered attractive producers of biotechnologically important enzymes due to their evolved systems for breaking down complex organic materials (Calusinska et al., 2020; Reichart et al., 2021; Sethupathy et al., 2021). The enzymes responsible for degradation of carbohydrates are called carbohydrate-active enzymes (CAZymes) and are present in virtually all living forms (Drula et al., 2022). Previous observations indicated that genomes belonging to the *Planctomycetota* phylum harbor a significant number of diverse CAZymes (Gharechahi et al., 2022; López-Mondéjar et al., 2022; Sun et al., 2023). For instance, *Planctomycetota* from AD reactors, but also peat-enriched wetlands are presumed to be well-equipped toward complex polysaccharide decomposition (Ivanova et al., 2018; Vanwonterghem et al., 2016). As expected, the genomes most likely to possess the greatest capacity for polysaccharide scavenging were primarily recovered from metagenomes of environments rich in organic matter (Klimek et al., 2024).

Since the discovery of *Planctomycetota*, numerous studies have documented their widespread occurrence and abundance across diverse environments. Members of this phylum have been detected in marine and brackish waters (Cabello-Yeves et al., 2021; Figueroa et al., 2021; Howe et al., 2023; Storesund et al., 2020), extreme habitats (Elshahed et al., 2007; Farias et al., 2017; Spring et al., 2015) including glaciers and permafrost (Malard and Pearce, 2018; Vipindas et al., 2020; Yang et al., 2016) and various terrestrial ecosystems (Buckley et al., 2006; Hu et al., 2022). Although primarily considered environmental bacteria, certain species, particularly within the *Gemmata* genus, have been linked to opportunistic infections in immunocompromised individuals (Aghnatios and Drancourt, 2016; Kaboré et al., 2020). Representatives of this phylum have also been found in caves (De Mandal et al., 2017; Shen et al., 2022) and as host-associated bacteria, including associations with phytoplankton (Bondoso et al., 2017; Kim et al., 2016; Vollmers et al., 2017), invertebrates (Eilmus and Heil, 2009; Richards et al., 2017; Sharma et al., 2014; Vargas-Albores et al., 2017), animals (Gharechahi et al., 2022; Tran et al., 2018; Yildirim et al., 2010), and within rhizosphere microbiomes (Nunes da Rocha et al., 2009; Ravinath et al., 2022). In built environments, studies of microbial communities in wastewater treatment plants frequently report the presence of *Planctomycetota*, particularly in relation to the anaerobic ammonium oxidation (anammox) process (Suto et al., 2017). Nevertheless, while the phylogenetic diversity of *Planctomycetota* inhabiting AD and peatlands were already analyzed (Dedysh and Ivanova, 2019; Klimek et al., 2025), their presence in the other biomass-rich habitats remains poorly understood.

To fill this gap, in this study, we aimed to explore and organize current knowledge on the phylogenetic diversity of *Planctomycetota* community across diverse biomass-rich environments. For that purpose, we compiled previously generated datasets from AD reactors, soil, and termite gut and nest samples, and complemented them with additional samples from habitats such as freshwater sediments, decaying biomass, wetlands, and rhizospheres or soil covered by cryptogams (e.g., lichens and mosses). All samples were further analyzed using 16S rRNA gene sequencing to assess the diversity of planctomycetotal operational taxonomic units (OTUs) and to examine whether these taxa are phylogenetically conserved or broadly distributed across the studied environments.

## Materials and methods

### Data collection and sample preparation

To assess the planctomycetotal diversity, we combined our previous amplicon sequencing data with the datasets newly generated in this study. Briefly, datasets for anaerobic digester microbial community were taken from Calusinska et al. (2018), while the samples from termite species were taken from Marynowska et al. (2020). Only a random selection of sequenced samples was used targeting between 5 and 20 samples per environment (74 in total, Supplementary Table S1). To complement the datasets with other biomass-rich environments, we additionally sampled different soil types, compost, peat-accumulated wetlands, freshwater sediments, decaying biomass and animal manure for amplicon sequencing (in total 45 more samples, Supplementary Table S1). Samples were collected using sterilized spatulas at the minimum of 3 cm depth material (e.g., soil, sediment, compost, manure) and were frozen if not processed the same day. The exact list of all the samples used in this study is detailed in Supplementary material.

### 16S rRNA gene amplification and sequencing

To generate 16S rRNA gene amplicons sequencing data of additionally sampled habitats, DNA of 45 samples were extracted from 0.5 g of sample material using PowerSoil kit (QIAGEN) following the manufacturer’s instructions. No technical replicates were prepared. The quality of extracted DNA was assessed using NanoDrop spectrophotometer. The 16S rRNA gene sequences were amplified using pair of custom primers (F-ACTCAAAKGAATWGACGG and R primer-ACGGGCGGTGTGTRC) targeting about 500 bp of the V6-8 region. The conditions for PCR cycling were as specified: initial denaturation 30s at 98°C followed by 22 cycles of denaturation 5 s at 98°C, annealing 30s at 58°C and 30s at 72°C of elongation. Final elongation step was performed at 72°C for 5 min. Amplicon library sequencing was prepared using Nextera XT DNA Library Preparation Kit and compatible set of indices (Nextera XT Index Kit v2), with addition of PhiX control at 5%. Paired-end sequencing was performed on an Illumina MiSeq instrument using v3 chemistry.

### 16S rRNA gene amplicon data processing

The newly generated amplicon sequencing data along with previous datasets were processed together. The data processing including paired reads merging, quality filtering as well as OTU clustering and construction, were undertaken using USEARCH v11.0.667. Paired-end reads were merged by using-fastq_mergepairs -relabel @ command. The primer sequences and distal bases were truncated from the merged reads with the following parameters: -fastx_truncate-stripleft 22, −stripright 15. Singletons and reads shorter than 400 bp were removed using-sortbysize and-fastq_maxee commands. Sequences which shared 97% similarity were binned into operational taxonomic unit (OTUs) with the UPARSE algorithm (−cluster_otus) using filtered reads. OTU count table was built by-otus command. Taxonomy was assigned to each OTU using “classify.seqs” command, implemented in mothur v1.48.0 by aligning to the SILVA 16S rRNA gene database v138 (Quast et al., 2013; Schloss et al., 2009). Taxonomical annotation was further used to retain all OTUs assigned to *Planctomycetota* (100%). Subsequently, OTU read counts were trimmed and samples with less than 100 planctomycetotal reads were removed from further analysis. To account for variation in sequence depth between sequenced datasets and samples, counts were normalized by subsampling each sample’s read counts to a median of the sample sums (median*x/sum(x)).

### Phylogenetic analysis of planctomycetotal community

Retrieved planctomycetotal OTU sequences were first aligned using muscle. Phylogenetic tree was then constructed in Geneious v2019.0.3 using Neighbor-Joining method. Additionally, Faith’s Phylogenetic Diversity (PD) index was used to quantify the total branch length of a phylogenetic tree that encompasses all taxa in a sample (implemented in picante R package; Kembel et al., 2010). Using mothur (unweighted.unifrac), UniFrac distances were also computed using pre-calculated tree in Newick format (Lozupone and Knight, 2005). The planctomycetotal OTU sequences were additionally blasted against a custom database containing 16S rRNA gene sequences retrieved from all the type strains of *Planctomycetota* described until August 2024 (blastn -max_target_seqs 1 -evalue^1e-5^).

### Statistical analysis of amplicon sequencing data

All analyses were carried out in R studio using the vegan package v2.6 (commands are detailed in brackets) and applied to a matrix containing the planctomycetotal normalized read counts (Oksanen et al., 2001). Alpha diversity, e.g., within-sample planctomycetotal diversity, was estimated by the number of observed distinct OTUs (richness) and the Shannon Index (diversity). Bray-Curtis and Jaccard dissimilarity indices were computed to quantify community composition differences across samples, e.g., beta-diversity (vegdist). To provide visual representation of sample clustering, the calculated dissimilarities were used for ordination analysis using non-metric multidimensional scaling, nMDS (metaMDS). The correlation between indices was assessed using Spearman’s rank correlation. To assess the significance of habitat differences, the Analysis of Similarities, ANOSIM (anosim) test was performed using Bray-Curtis distances with 1,000 permutations. Additionally, permutational multivariate analysis of variance (PERMANOVA) was applied to evaluate statistical significance of group differences (adonis2). The significance of differences between environments was assessed using pairwise PERMANOVA (pairwise.adonis package). Differences in alpha diversity indices (Shannon and Faith’s Phylogenetic Diversity) across environments were assessed using the Kruskal-Wallis test, followed by pairwise Wilcoxon rank-sum comparisons with false discovery rate correction for multiple testing. For equality of variances testing, the homogeneity of group dispersions was validated (betadisper). Finally, the Multi-Response Permutation Procedure (MRPP) was conducted to further test for significant differences between habitats (mrpp). To test for the presence of differentially abundant OTUs and genera between significantly different samples and environments, both similarity percentage (SIMPER, implemented in vegan) and Linear discriminant analysis Effect Size (Lefse) analyses were applied. Lefse was calculated using mothur (lefse) and the threshold for the linear discriminant analysis score was set to 2.0.

## Results

### Sampled habitats and repository data used in this study

**Figure 1:**
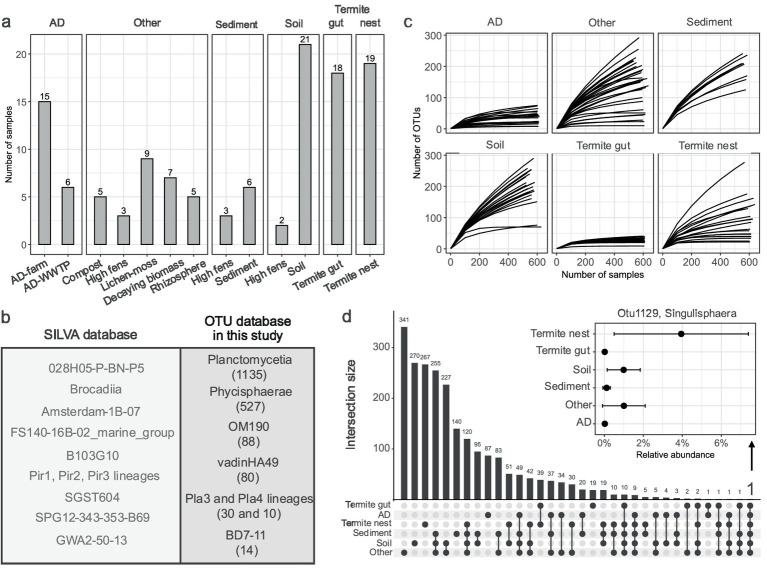
Studied environments and recovered OTUs of *Planctomycetota*. **(a)** Number of samples considered for the analysis, distinguished by the main type and sub-type environment categories. **(b)** Comparison of SILVA taxonomy of retrieved OTUs to all the other known SILVA taxonomy. Left box: taxa in SILVA not found in this study. Right box: taxa in SILVA found in this study, in brackets number of detected OTUs assigned to each class. **(c)** Rarefaction curves showing OTU richness across habitats, illustrating the relationship between sequencing depth and microbial diversity. **(d)** UpSet plot representing the intersection of shared and unique OTUs across different samples. Additionally, the relative abundance of one common OTU for all the environments (Otu1129) grouped in main type environments is depicted.

Throughout the study, we use the term biomass-rich environments to describe habitats that we analyzed, characterized by high levels of primary and secondary organic plant matter. These environments included diverse representative niches sampled in Luxembourg, Belgium and French Guiana (Supplementary Table S1). To simplify the analysis, we split the dataset of samples into two environment type categories. Main type included AD, soil, freshwater sediment, termite nest (mound), termite gut and all the other environments. Sub-type environment categories included AD (AD-WWTP from wastewater treatment plant fed with sewage sludge and AD-farm coming from agriculture-and biowaste-fed reactors), soil (soil including arable, grassland and forest, rhizosphere, moss-lichen covered and high fens), compost, decaying biomass (algal bloom, fungus-infected trees, biological mat), freshwater sediment, termite nest and termite gut. Three animal manure samples (two cattle and one horse) were collected, but due to the low number of planctomycetotal reads, only one sample (horse manure) was included in the analysis (Figure 1a, not shown). The samples taken from wetlands were also grouped together into “high fens” category when needed (Supplementary Table S1; Figure 1). Although our study did not include technical replicates, repeated measurements of the same environment type (biological replicates) should provide sufficient consistency in the data.

### The amplicon sequencing output and SILVA taxonomy comparison

The sequenced dataset from this study included 45 samples, producing 8.7 M of read pairs. The initial quality check revealed an average read length of 484 bp, with a base quality score consistently above 30. Most of the reads passed the quality filtering (5.9 M), and we obtained a total of 1.7 M of unique, high-quality sequences remained for downstream analysis. The sequences were next combined with the reads from previous datasets yielding a final dataset of 119 samples (Supplementary Table S1; Figure 1a). OTU clustering of all the sequences with singletons removal resulted in generation of 26,578 OTUs, including a total of 1,899 OTUs that belonged to the *Planctomycetota* phylum, contributing to about 7% of the total bacterial diversity. At first, to assess the diversity within the studied environments, we classified retrieved OTUs against the SILVA database, comparing the detected taxonomic classes to those known in SILVA to identify the extent of overlapping class-level diversity (Figure 1b). Of all known classes and orders within the *Planctomycetota* phylum cataloged in the SILVA database, more than half were detected in this study, highlighting the broad diversity present in biomass-rich environments. Taxonomic classification of the identified OTUs revealed the presence of nine putative and known classes of *Planctomycetota*. Specifically, 1,135 OTUs were assigned to *Planctomycetia*, while 527 to *Phycisphaerae* classes. Next, putative classes such as OM190, vadinHA49 and Pla4 lineages were represented by 88, 80, and 30 OTUs, respectively. Fewer than 15 OTUs were found to be assigned to the Pla3-lineage, BD7-11, ODP123, and *Ca.* Brocadiia. The rarefaction curves for each sample reached a plateau only for AD, termite gut and certain other sub-type environments, indicating that sequencing depth was sufficient to capture the majority of the planctomycetotal diversity only for these systems (Figure 1c).

### Shared and unique planctomycetotal OTUs among habitats

The majority of OTUs were shared between soil, sediment and other type environments, with the largest intersection of 255 OTUs observed, indicating significant phylogenetic overlap between these ecosystems (Figure 1d). In contrast, a smaller subset of 19 OTUs was uniquely found in termite gut samples, reflecting the specialized nature of the microbial community associated with this environment. The AD reactor samples also shared a considerable number of OTUs (49) with the soil, sediment and other category samples, which may be indicative of the transfer of microbes through organic waste inputs used in anaerobic digestion process. Only one OTU belonging to MSBL9-clade (*Sedimentisphaerales*) was found to be common between the AD and termite gut sample sets (Otu28617). The smallest intersections involved termite gut, which shared relatively few OTUs with other environments (10). Only one OTU, e.g., Otu1129, assigned to the *Singulisphaera* genus, was evidenced to be present in all the environments studied and it shared 99.1% sequence identity to the 16S rRNA sequence of *Singulisphaera rosea* S26. Importantly, the relative abundance of Otu1129 in the environments other than soil and termite nest (on average between 1.5 and 4%) was negligible, e.g., 0.2%, 0.2–0.5, and 0.5% of planctomycetotal abundance in gut, sediment and AD (Figure 1d).

### *Planctomycetota* relative abundance and alpha diversity in studied environments

**Figure 2:**
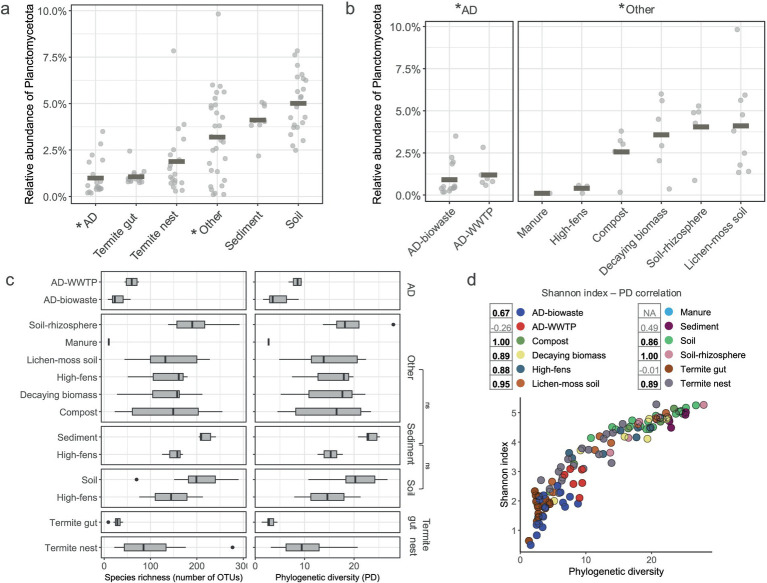
Alpha-diversity of *Planctomycetota* communities. **(a,b)** Relative abundance of Planctomycetota in samples grouped by **(a)** the main type environments **(b)** and sub-type environments. Gray rectangles indicate the mean abundance **(c)** Planctomycetotal OTU richness (number of distinct OTUs) and phylogenetic diversity (Faith’s PD) for all the sub-type environments; ns, non significant. **(d)** The correlation between SD and PD indices of all the studied samples, colored by sub-type environment categories. Numbers indicate Spearman rank rho correlation values and significant correlations (*p* < 0.05) are denoted in bold. Manure is represented by only one sample and therefore no correlation was calculated.

The *Planctomycetota* phylum was observed with abundances ranging from nearly 0% to almost 10% in the studied samples (Figure 2a). Looking at the individual samples, the highest abundances were detected in lichen-moss soil cover (9.82%) and termite nest (7.8%) samples and the lowest in horse manure specimen (Figure 2b, 0.1%). On average, soil samples contained the highest abundance of *Planctomycetota* (5.2% ± 1.4), followed by the sediment and other soil-types samples (Figure 2b), resulting in 4.2% (±1.1), 4.1% (±2.8), and 4.0% (±1.8), respectively. In soil samples, the higher relative abundance of *Planctomycetota* can be attributed to the cumulative presence of diverse OTUs at low concentrations (Supplementary Table S3). In turn, the lowest average planctomycetotal abundance was found in AD reactors (0.91% ± 0.99) and termite gut (1.1% ± 1.4) habitats.

To characterize the planctomycetotal diversity within each sample, we assessed the Faith’s phylogenetic diversity (PD) and richness (observed OTUs) across habitats (Figures 2c,d). Both indices varied significantly among the studied environments (*p* < 0.01). However, pairwise comparisons between habitats revealed that soil, sediment, and “other” types of environments exhibited similar SD values, as indicated by non-significant pairwise comparisons (Figure 2c). Soils and sediments exhibited the highest PD (23.3 ± 1.7 and 20.3 ± 4.2, respectively) indicating a broad evolutionary span of the planctomycetotal community. In contrast, termite guts showed the lowest PD (3 ± 0.8), suggesting more evolutionarily narrow community. AD samples showed higher discrepancy of planctomycetotal diversity, and AD-WWTP had higher PD (8.4 ± 1.1) than AD-farm (4.5 ± 2.2). Similar patterns occurred when analyzing the species richness, with the highest number of observed OTUs in soil and sediment samples. We next calculated the Shannon diversity index (SD) and compared it with the PD value for each sample (Figure 2d). Spearman correlation analysis between these indices indicated a significant positive correlation (*p* < 0.05) for most of the habitats, suggesting a higher number of observed OTUs and the evolutionary breadth of planctomycetotal communities. In contrast, the habitats with lower PD, such as termite guts and ADs, were also characterized by lower species richness. The comparison between PD and SD showed that, while AD-WWTP had higher species diversity, the negative correlation between these two indices suggests that the taxa may be more closely related, despite their OTU abundance. Taken together, this indicates that the microbial communities in both AD-WWTP and the termite guts are more specialized.

### Beta diversity of planctomycetotal communities among studied environments

**Figure 3:**
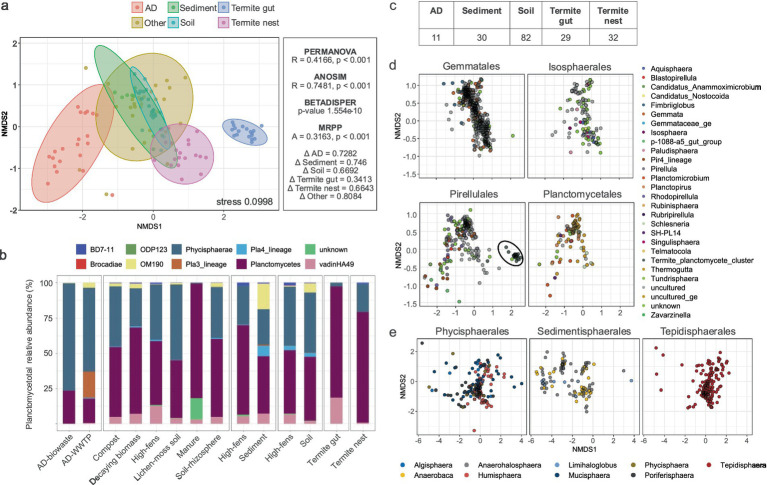
Beta diversity of *Planctomycetota* communities. **(a)** nMDS plot illustrating planctomycetotal community composition across diverse environments based on Bray-Curtis dissimilarities. Right panel presents the results of beta diversity statistical tests. **(b)** Barplot represents the planctomycetotal community for class level as assigned against SILVA database. **(c)** Number of differentially abundant OTUs in each of the environments. **(d)** nMDS plot for the orders within the *Planctomycetia* class, colored by genera; in black ellipse the termite cluster is highlighted. **(e)** nMDS plot for the orders within the *Phycisphaerae* class, colored by genera.

Subsequently, we examined differences in the planctomycetotal community composition between studied environments. nMDS plot using Bray–Curtis dissimilarity revealed distinct clustering patterns based on the sampled habitat, especially visible for planctomycetotal communities in anaerobic digesters and termite guts (Figure 3a). The nMDS analysis yielded a stress value of 0.0998, indicating a good representation of the data. The PERMANOVA test confirmed that these differences in community composition were statistically significant (Figure 3a), with pairwise PERMANOVA revealing significant differences between all habitat types except among sediment, soil, and the “other” category environments. However, the significant betadisper result suggests that part of the difference detected by PERMANOVA could also be influenced by differences in how dispersed the community compositions are within each habitat. We also confirmed that the clustering patterns indicated only termite gut as a more homogenous community (delta of 0.34), while all the other environments showed higher variability (delta > 0.66). It is important to note that delta values may vary depending on multiple factors; therefore, these thresholds should be interpreted within the context of this dataset and not as universal cutoffs. Overall similarity index scored to 0.316 supporting a moderate to strong agreement within groups, meaning that the samples within each habitat were more similar to each other than what would be expected randomly (Figure 3a). Across all samples, not surprisingly, the most abundant classes were *Planctomycetia* and *Phycisphaerae*, constituting at least one third of total sequences (Figure 3b). Representatives of other putative planctomycetotal classes such as OM190 (proposed Saltatorellus) and vadinHA49 were also found to be in higher abundances in sediments and termite gut samples. Certain *Planctomycetota*, such as Pla3 (UBA8108 in GTDB) and other yet-unknown lineages, were detected almost exclusively in AD-WWTP and manure samples, suggesting a possible niche specialization (Figure 3b).

To identify taxa that were differentially abundant between habitats, SIMPER and LEfSe analyses were employed (Figure 3c). As a result, we identified 178 OTUs significantly different among all main type habitats, belonging primarily to 50 genera. The highest number of differentially abundant OTUs was found in soil (82), but representing only 7.8% of all the OTUs discovered. On the other hand, 40% of all the OTUs found in termite guts were differentially abundant. We next tried to visualize potential co-occurrence patterns among taxa and performed nMDS based on taxonomic distributions. As a result, we observed that *Gemmatales* and *Isosphaerales* tended to overlap, as did *Planctomycetales* and *Pirellulales* (Figure 3d). Additional, clear grouping of termite-derived *Pirellulales* can also be observed (Figure 3d) Among *Phycisphaerae*, certain genera of *Phycisphaerales* and *Tepidisphaerales* tended to collocate, while *Sedimentisphaerales* showed clearer groupings (Figure 3e). In the following section we evaluated the genera that contribute the most to the patterns observed.

### The closest relatives of OTUs to known species

**Figure 4:**
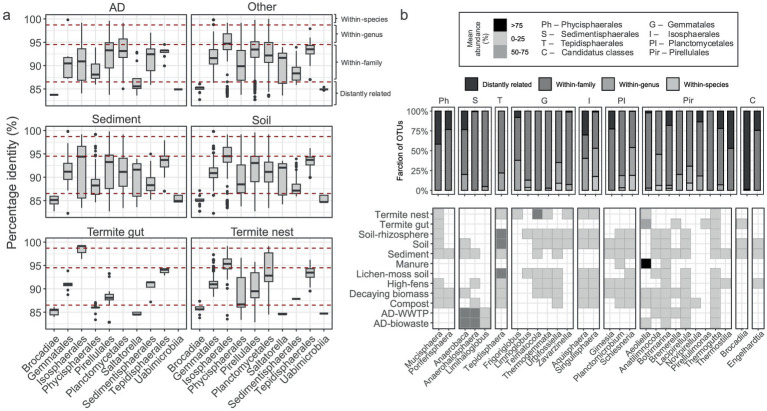
Comparison of *Planctomycetota* OTU sequences to the formally described type strains. **(a)** Percentage identity (%) for individual OTUs gathered at the order level; red lines limit the set thresholds for species, genus, family delineation. **(b)** The significantly different genera among all the studied environments. Barplot represents fraction of OTUs assigned to each thresholds category. Heatmap represents planctomycetotal average abundance range in each habitat (legend in the top box).

To gain a deeper ecological understanding of *Planctomycetota*, we compared their OTUs against a custom database containing formally described type species. The dataset was then stratified to identify potential OTUs matching known species, genera, and families, using standard identity thresholds of 98.7, 94.5, and 86.5% (Figure 4a). The majority of the retrieved OTUs in this study, e.g., between 22 and 60%, represent potential new genera within known families, depending on the lineage and its environmental origin (Supplementary Table S2). In turn, 49 OTUs exhibited high identity, potentially representing known species (Supplementary Table S2). Among them, four were commonly found in soils, termite nests and sediments: *Caulifigura coniformis* Pan44 (Otu774) (Kallscheuer et al., 2020b), *Schlesneria paludicola* MPL7 (Otu3995) (Kulichevskaya et al., 2007), *Paludisphaera soli* JC670 (Otu12607) (Kaushik et al., 2020) and *Singulisphaera rosea* S26 (Otu1129) (Kulichevskaya et al., 2012). The other typical soil-sediment OTUs with high identities were: *Urbifossiella limnaea* ETA_A1 (Otu5302), *Humisphaera borealis* M1803 (Otu2624) (Dedysh et al., 2021) or *Anatilimnocola aggregata* ETA_A8 (Otu3323) (Kallscheuer et al., 2021). In turn, in AD samples with high identities to known species were *Posidoniimonas polymericola* Pla123a (Otu5525) and *Planctomyces bekefii* (Otu3705) (Dedysh et al., 2020). Subsequently, we applied the Kruskal-Wallis test to identify genera with significant abundance changes across main environmental types, revealing a panel of 28 genera (Figure 4b). Most of these genera were not highly abundant in the studied environments, contributing between 0 and 25% to the planctomycetotal community. Only *Aeoliella*-assigned OTUs were highly abundant in manure sample representing >75% of the planctomycetotal community, while Anaerobaca and *Anaerohalosphaera*, both retrieved from ADs, were moderately abundant in these environments (between 50 and 75%). Tepidisphaera was also dominant *Planctomycetota* in diverse soils including rhizosphere and lichen-moss covered soils. *Ca.* Brocadiia-, *Thermostilla-and Mucisphaera*-related OTUs were identified with a high portion of distantly related 16S rRNA gene sequences*. Frigoriglobus* seemed to be a hallmark genus for the termite nest samples, and about 30% of its sequences expressed high identity to *Frigoriglobus tundricola* PL17 (Kulichevskaya et al., 2020). However, termite nest is most abundantly inhabited by OTUs potentially attributed to *Telmatocola*-related representatives (50 and 75%). *Thermogutta*-related OTUs were widespread in different habitats, however, they were also largely represented by within-family sequences. In turn, both *Singulisphaera-and Schlesneria*-assigned OTUs were represented by a high fraction of sequences with higher identities, e.g., passing genus threshold.

### Planctomycetotal shared community

**Figure 5:**
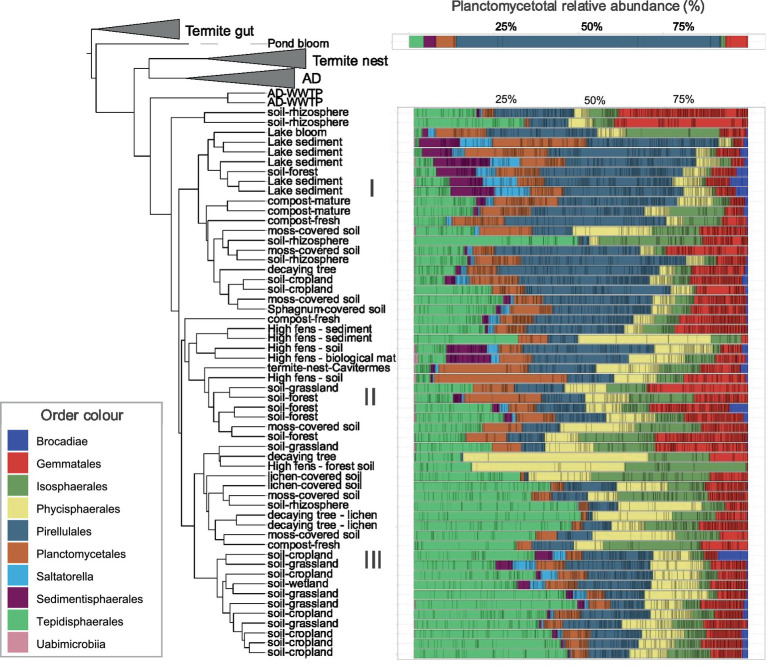
Shared *Planctomycetota* phylogeny for soil, sediment and other environment type samples. Weighted UniFrac phylogenetic tree illustrates the relationships between samples based on their planctomycetotal community composition, alongside corresponding barplots show the relative abundance of taxa at the order level.

The phylogenetic tree based on UniFrac distances showed that samples from termite guts, termite nests and AD (except two WWTP samples) formed three separate clusters, indicating strong planctomycetotal community structures specific to these environments (Figure 5). In contrast, samples from diverse soil types, sediments, composts and decaying biomasses showed mixed clustering patterns, often more influenced by specific local conditions (e.g., High-fens region) rather than habitat type. The statistical differences between these environments were indeed not significant, further strengthening the observation of similar community structure. Regardless, three clusters on the tree are visible: (I) comprising diverse soils, decaying biomass and sediment samples, (II) encompassing forest soil and High fens samples and (III) mostly consisting of soil samples. In cluster I, *Pirellulales* dominated the community, but sediment samples also comprised Saltatorellus and *Sedimentisphaerales*. Cluster II was mainly abundant in *Planctomycetales*, and *Gemmatales*, while cluster III was dominated by *Tepidisphaerales*. Pond bloom sample diverged remarkably from all the others, exhibiting a highly distinct community dominated by *Pirellulales* OTUs with approximately 80% of total relative abundance (Figure 5). Next, we identified a panel of shared OTUs that significantly contributed to the observed community structure of soil, sediment and other type habitats, reflecting the presence of mixed planctomycetotal communities. A total of 29 shared OTUs was assigned to *Tepidisphaera*, while other OTUs were assigned to *Telmatocola* (19), *Thermogutta* (18), *Anatilimnocola* (16), *Engelhardtia* (12), *Mucisphaera* and *Anaerobaca* (both 11) and *Humisphaera* (10).

### Common and specific lineages in different environmental stages

**Figure 6:**
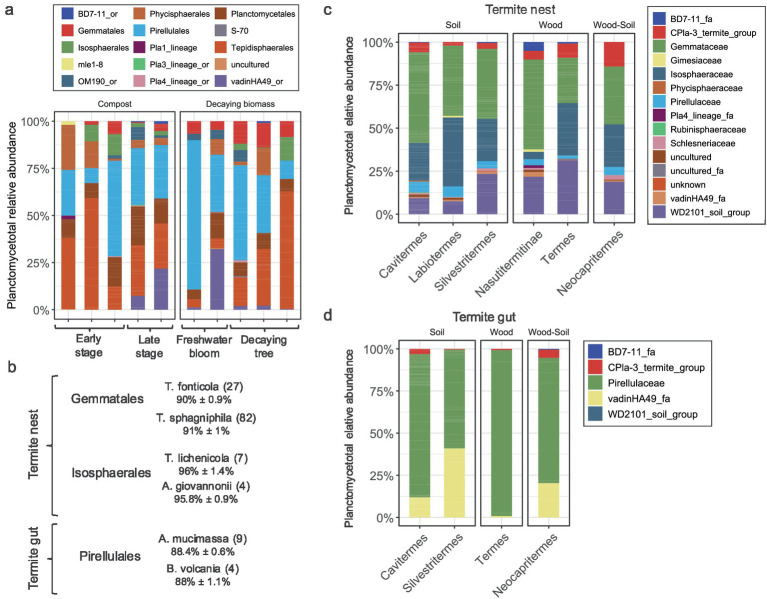
Phylogenetic affiliation of *Planctomycetota* for selected environments. **(a)** Relative abundance of *Planctomycetota* in actively degrading biomasses such as compost, freshwater blooms and decaying trees. **(b)** Percentage identity of the most abundant OTUs assigned to the main *Planctomycetota* orders: *Gemmatales* and *Isosphaerales* in termite nests and *Pirellulales* in termite guts. Shortcuts: *T. fonticola* (*Thermogemmata fonticola* 2918), *T. sphagnifila* (*Telmatocola sphagniphila* SP2), *T. lichenicola* (*Tundrisphaera lichenicola* P12), *A. giovannonii* (*Aquisphaera giovannonii* OJF2), *A. mucimassa* (*Aeoliella mucimassa* Pan181) and *B. volcania* (*Bremerella volcania* Pan97). In brackets the number of OTUs assigned to these strains are indicated. **(c,d)** Termite nest **(c)** and gut **(d)** planctomycetotal community. Relative abundance of different families of *Planctomycetota* are gathered for each species of termites.

In compost samples the community changed from early stage to more digested matter (Figure 6a). The vadinHA49 class was more commonly found in later stages of composting, e.g., in well-digested material. The vadinHA49 clones retrieved in this study were distantly related to other known, described *Planctomycetota* with average percentage identity below 85%. Blasted 16S rRNA gene sequences of vadinHA49 against deposited sequences in GTDB revealed that this group represents SZUA-567 putative class. Comparison of two different freshwater blooms showed significant differences in planctomycetotal communities (Figure 6a). Even though both samples taken from freshwater cyanobacterial blooms were sampled during its density peak leading to decomposition, pond sample was abundant in *Pirellulales* (Figure 6a), while bloom occurred on lake was more diverse, including other bacteria such as vadinHA49. Samples from decaying trees also did not show strong common patterns in relative abundance of *Planctomycetota*, however, *Tepidisphaerales* and *Pirellulales* seemed to be the dominant groups in these ecosystems. Except freshwater bloom samples, all the sampled environments seemed to be dominated by the same orders such as *Tepidisphaerales, Pirellulales* and *Planctomycetales.*

### Planctomycetotal community in termite bodies and nests

The *Planctomycetota* community in termite nests was distinct from soil samples, although there were overlaps, mainly driven by the presence of specific OTUs distantly related to *Gemmatales* and *Isosphaerales* (Figure 6b). Overall, the main distinction between the soil and termite nest planctomycetotal communities lies in the presence of termite-specific microbes, which shape the community structure and make it distinct from the typical soil members (Figures 5, 6c). Looking at the different feeding styles of termites, the wood-feeding termite nests showed higher richness (158 ± 128 OTUs), compared to nests of termites feeding on soil humus (84 ± 44 OTUs) and mixed humus-wood feeders (40 OTUs). Conversely, OTU richness in termite guts was the highest in humus-feeders (30 ± 6 OTUs), rather than in wood-and mixed-feeders (20 and 9 OTUs, respectively). Termite guts were chiefly dominated by OTUs assigned to *Pirellulaceae* and vadinHA49 families, except a single sample of wood-feeder, consisting of only 9 *Pirellulales* OTUs (Figure 6d). Otu993 seemed to be a core termite gut representing Termite_planctomycete_cluster in SILVA database, being present in all the termite species studied. Otu993 displayed the highest 16S rRNA gene similarity to *Aeoliella mucimassa* Pan181 of 88.5% suggesting the potentially novel sister family to *Lacipirellulaceae* (Wiegand et al., 2020) (Figure 6b).

## Discussion

In the context of biomass degradation, microbial community analyses are essential not only for revealing taxonomic diversity but also for providing insights into possible ecological functions and guiding future microbial isolation efforts. Therefore, the aim of this study was to characterize the microbial community of the *Planctomycetota* phylum based on 16S rRNA gene sequencing, providing insights into their diversity and composition across diverse natural and built biomass-rich systems. We revealed significant variations in *Planctomycetota* community composition that reflect common patterns found in microbial communities globally. The OTU rarefaction curves showed contrasts in *Planctomycetota* richness across environments (Figure 1) and disclosed OTU diversity across habitat types that was further supported by alpha diversity metrics (Figure 2). Commonly, microbial communities from soil, followed by sediment, show the highest alpha diversities (Walters and Martiny, 2020), and this is well reflected by *Planctomycetota* richness and diversity in these environments. Microbes from habitats such as anaerobic digesters or termite guts are predicted to exhibit narrow OTU diversity, as both communities are highly specialized for the specific anaerobic niches present in these environments (Goux et al., 2023). Consequently, the observation of a limited number of planctomycetotal OTUs is not unexpected. In some environments, the total planctomycetotal abundance is negligible, approaching levels typically associated with the rare biosphere (below 1% or even lower), while in other environments, the greater diversity of species parallels the higher abundance. Despite this variability, in both cases, *Planctomycetota* could serve as potential genetic reservoirs, harboring unique genetic diversity that may contribute to maintaining the functional resilience of the entire ecosystem.

The species delineation and estimation are still challenging due to the limited resolution of 16S rRNA gene amplicon sequencing or influence of primer selection, which can lead to misclassification and inaccuracies in estimating microbial diversity (Abellan-Schneyder et al., 2021; Johnson et al., 2019). In this study, OTU clustering was performed at the 97% sequence similarity threshold, a widely used approximation for species-level diversity. While higher-resolution approaches, such as 99% OTU clustering or amplicon sequence variants, are proposed as the current standard (Callahan et al., 2019; Edgar, 2018), 97% OTU clustering still offers certain advantages. In our study, such approach allowed comparability across datasets originating from different sequencing runs, helped reduce the impact of sequencing noise or artifacts and importantly, lowered the risk of subdividing taxonomic units into poorly supported taxa, resulting in more conservative and robust taxonomic assignments (Schloss, 2021). Still, future work employing full-length 16S rRNA gene sequencing may offer a more accurate view of *Planctomycetota* diversity.

Surprisingly, one OTU with high identity to *Singulisphaera rosea* S26 belonging to the *Isosphaeraceae* family (*Isosphaerales* order) was shared among all the studied habitats, but only abundant in diverse soil types, termite nests and other type environments including composts or high fens (Figure 1). Previously, *Planctomycetota*, including *Isosphaerales*, were indeed found to represent a significant fraction of the microbial communities in lichen-dominated areas, contributing to 8–13% of total bacterial sequences (Ivanova et al., 2016). Isolated *Singulisphaera* strains from these habitats were also shown to utilize complex polysaccharides including a characteristic component of lichen biomass (lichenan), but overall *Singuliphaera* spp. strains are capable of utilizing a wide range of polysaccharides including cellulose and chitin (Kulichevskaya et al., 2012; Ivanova et al., 2024). The widespread presence of *Singulisphaera* OTUs suggests its potential metabolic versatility, making it a compelling subject for further study. This is particularly important in light of its metabolic plasticity as members of the *Isosphaerales* order are also known to harbor plasmids (Coronel et al., 2024; Ivanova et al., 2017).

The high relative abundance of *Pirellulales* and *Tepidisphaerales* in several habitats including soils, sediments, freshwater blooms and animal guts suggests that these microbes play substantial roles in the studied environments, including putative breakdown of biomass. This degradation process is likely driven by fermentative and hydrolytic pathways, as described species from both orders are heterotrophic bacteria possessing multiple CAZymes, which certainly facilitate the breakdown of complex organic matter (Klimek et al., 2024). For instance, previous study indicated that *Tepidisphaera* was involved in degradation of extrapolymeric substances in biochar-assisted bioremediation reactors (Jiang et al., 2020). The planctomycetotal community in the pond bloom sample was dominated by three *Pirellulales* OTUs accounting for approximately 80% of the total relative abundance (Figure 5). The OTUs identified had the highest similarities of 95.3% to *Aureliella helgolandensis* and 95.1 and 94.3% to *Bremerella alba* FF15. Both strains were isolated from host-associated environments as they derived from jellyfish and macroalgae ecosystems (Godinho et al., 2021; Kallscheuer et al., 2020a). This highly specialized community aligns well with previous observations, as members of the Pirellulales were shown to proliferate during cyanobacterial blooms in freshwater environment, where they became highly abundant, albeit not particularly transcriptionally active (Kallscheuer et al., 2021). In turn, *Planctomycetota* assigned to *Pirellulales*, were also evidenced as the most transcriptionally active group of microbes in the hydrothermal plume, involved in organic matter scavenging (Li et al., 2016).

Based on the set thresholds for taxonomic delineation, the OTUs recovered in this study were primarily associated with already known bacterial families (Figure 4). These families are generally represented by cultured type species, suggesting that most discoveries in biomass-rich environments will likely involve new genera within the established higher ranks. However, some yet-unknown lineages likely remain undescribed. All OTUs distantly related to Brocadiia suggest existence of new class-level lineages of *Planctomycetota* specific to soils, sediments, but also termite guts. One of the lineages of putative class status is vadinHA49 (in SILVA, SZUA-567 in GTDB). This group has been previously identified in various environments, including anaerobic digesters, the intestinal tracts of insects, peat bogs, and sulfur spring sediments (Ivanova et al., 2018; Sabree and Moran, 2014; Youssef et al., 2012). It was also evidenced that vadinHA49 strongly correlated with the higher availability of organic matter, suggesting its involvement in biomass degradation during AD (Liu et al., 2020). In addition to that, vadinHA49 was also identified as one of the bacterial groups able to persist the cadmium pollution (Song et al., 2019). However, studies on vadinHA49 are scarce, and its potential metabolic functions remain of high interest, though they are largely unexplored. *Thermogutta* exhibited differential abundance across the studied environments, simultaneously with many OTUs related to this genus shared among soil, sediment, and other habitat types. Additionally, their lower sequence similarities to members of the *Thermoguttaceae* family suggest that they represent a novel lineage within this family. *Thermoguttaceae* representatives inhabit terrestrial, subsurface or anthropogenic ecosystems with elevated temperatures (Slobodkina et al., 2015, 2016); yet our analysis clearly shows their widespread occurrence in other ecosystems. Previously, we identified *Thermoguttaceae* as putative biomass degraders due to the presence of multiple CAZymes (Klimek et al., 2024). Therefore, the wide distribution of these novel lineages across the studied biomass-rich environments is not unexpected.

Soil, sediment and other type environments shared certain widespread taxa (Figure 5) distantly affiliated to *Tepidisphaera* (*Tepidisphaerales*), *Telmatocola* (*Gemmatales*), *Thermogutta*, and *Anatilimnicola* (both *Pirellulales*). As previously noted for the *Isosphaera* genus, the genera listed may also exhibit metabolic versatility that enables adaptation to diverse environmental conditions. Despite pronounced physicochemical differences among habitats, these planctomycetotal groups may fulfill similar ecological functions, contributing to a convergence in core community composition. Future comparative genomic analyses of representative genomes from these genera will be instrumental in elucidating their metabolic capabilities and ecological roles. *Planctomycetota* are important and diverse components of soil microbial communities, and their diversity correlates strongly with environmental heterogeneity, particularly in relation to organic and nitrate levels as well as land use history (Buckley et al., 2006). Phylotypes belonging to *Gemmata* were previously linked to N_2_O reduction in soils (Xiao et al., 2025) and were also found to be the most abundant genera in the microbial community derived from garden soil that was actively removing the heavy metal pollutions (Singh et al., 2022). As also evidenced in our study, *Gemmatales* were previously listed among abundant orders in termite nests and suggested as an indicator of elevated phosphorus content in soil (González Plaza and Hradecký, 2023). Still, the abundance of *Planctomycetota* in the termite nest are usually lower than in the surrounding soil (Chen et al., 2021).

Interestingly, two trends of planctomycetotal beta diversity could be observed (Figure 3). Both *Gemmatales* and *Isosphaerales* tended to overlap in their community distribution, likewise *Planctomycetales* and *Pirellulales*, most likely due to their preferred environmental niches. The former two are more frequently associated with terrestrial and freshwater systems, in contrast to *Planctomycetales* and *Pirellulales*, which representative strains were typically isolated from marine, brackish or other aquatic environments (Vitorino and Lage, 2022). This is, however, more likely a result of their close phylogenetic relatedness, as phylogeny can exhibit a strong signal for habitat preferences, though this does not necessarily imply phylogenetic niche conservatism (Barberán et al., 2014). Based on the 16S rRNA gene phylogeny, *Isosphaerales* and *Gemmatales* are positioned on separate branch, indicating they are more distantly related to *Pirellulales* and *Planctomycetales* (Vitorino and Lage, 2022).

Animal gut habitats exhibited unique planctomycetotal assemblages predominantly affiliated with the *Pirellulales* order (Figure 5). The low abundance of *Planctomycetota* in the studied manure sample is consistent with previous reports on their presence in horse gut (Mach et al., 2022). However, 20% of OTUs reconstructed in this study could not be classified within any group in the SILVA database. Their percentage identity to *Aeoliella mucimassa* Pan181 (*Lacipirellulaceae; Pirellulales*) was oscillating around 88.3% ± 0.8%, indicating the presence of potentially novel lineages that may be specifically associated with the gastrointestinal tract of animals. Although our study lacked sufficient *Planctomycetota* reads in cattle manure samples, there is substantial evidence supporting their presence and diversity in the cattle rumen and ruminants overall. This is demonstrated by the abundance of assembled genomes from gut microbiome studies and their sporadic detection in various scientific reports (Hagey et al., 2019; Kim et al., 2014; Mao et al., 2012; Stewart et al., 2019). The relative abundance of Planctomycetota in the rumen is generally low, typically below 0.1%; however, studies have shown a significant increase in their abundance in grass-fed cattle and older animals (Liu et al., 2022; Wang et al., 2021). Samples collected from a diverse range of animals, including mammals, birds, fish, and insects, are predominantly inhabited by *Pirellulales*, suggesting a highly adaptable, host-associated planctomycetotal group (Medina-Silva et al., 2018). The ecological role and distribution of this bacterial group within the human gut microbiome remain unresolved. To date, *Planctomycetota* were only identified in a single 16S rRNA gene-based study, derived from stool samples of individuals in France and Senegal (Cayrou et al., 2013). Additionally, a limited number of genomes have been recovered from gut samples of hunter-gatherer populations (Carter et al., 2023). Previously, we noticed that *Pirellulales* express the potential to degrade mucosal layer components (mucins) from animal tissues such as the gastrointestinal tract (Klimek et al., 2025), presenting a promising avenue for future research. In addition to potential of *Pirellulales* for dietary and host polysaccharide degradation, they may also play other ecological roles within the gut ecosystem. *Pirellulaceae*-related lineages increased their abundance in cattle fed with grass infected with toxic endophyte *Epichloë coenophialum*, likely due to their ability to degrade main alkaloid (ergovaline) produced by this fungus (Mote et al., 2019). *Pirellulaceae* have the potential for degradation of hydrocarbon, and are resistant to pollutants (de Araujo et al., 2021; Flores et al., 2014), which may allow them to contribute to bioremediation processes. In general, other *Planctomycetota* from yet unidentified lineages have also been suggested as potential heavy metal detoxifiers, as their abundance was previously found to positively correlate with elevated cadmium and lead concentrations (Li et al., 2022).

Another interesting gut microbiome is that of termites. Termites play essential roles in ecosystems by decomposing plant material and contributing to nutrient cycling, ultimately returning organic matter to the soil (Ohkuma and Brune, 2011). Termite microbial communities are often inhabited by host-specific lineages (Arora et al., 2022). So-called lower termites are mainly wood-feeders that help in breaking down dead wood, while higher termites, mainly belonging to the *Termitidae* family, are characterized with wider food repertoire (Brune, 2014). Still, most of the higher termites ingest humus-rich soil, which contains decayed organic material, plant debris, and minerals. Lower termites represent the ancestral lineages that rely on protozoa for cellulose digestion, in contrast to higher termites, which have evolved a fully bacterial gut with a complex hindgut (Marynowska et al., 2023). The presence of *Planctomycetota* MAGs across a wide range of termite species supports the notion that this phylum constitutes a core component of the termite gut microbiome (Hervé et al., 2020), despite their lower abundancies. The ecological role of *Planctomycetota* in this environment remains purely hypothetical and is primarily inferred from genomic data. So far, *Planctomycetota* were suggested to increase the solubility of humic substances or to be involved in the digestion of microbial polymers (Köhler et al., 2008). Still, many plantomycetotal MAGs recovered from metagenomes from both lower and higher termites were found to be abundant in genes coding for pectinases (Salgado et al., 2024), implying they are likely involved in the degradation of plant organic matter. The current understanding of *Planctomycetota* abundance across different gut regions remains fragmentary. Their higher abundancies were found in the alkaline posterior compartments of hindgut in the soil-feeding termites belonging to the *Cubitermes ugandensis* and *Ophiotermes* sp. Ox79b (Köhler et al., 2008; Mikaelyan et al., 2017). In our study, the entire gut was taken for analysis, without separating it into compartments. Despite this, our findings, along with prior studies, indicate that *Planctomycetota* are not exclusively linked to higher termites nor alkaline conditions (Abdul Rahman et al., 2015). For instance, *Planctomycetota* were also found abundant in the foregut of the lower termites like the wood-feeding *Coptotermes formosanus* (Dar et al., 2024). Further investigation into the exact differentiation between planctomycetotal species in lower and higher termites would help elucidate their roles in either early-stage carbohydrate metabolism to lignocellulose degradation and fermentation. Counterintuitively, we observed higher richness and diversity of *Planctomycetota* in humus-feeders than in wood-feeders, indicating presence of a few, but specialized microbes for targeting lignocellulose. These novel, termite-specific bacterial lineages are uniquely adapted to the environment, reflecting observed tight clustering of termite gut samples. Termite gut and ADs systems are indeed characterized by distinct microbial communities, yet much of the encoded CAZyme diversity is shared among their microbes (Goux et al., 2023). Overall, future comparative genomic analysis of termite gut with gastrointestinal systems of ruminants will help identify specialized *Planctomycetota* essential for lignocellulose breakdown.

## Data Availability

All the results presented in this study are included in the article and its related Supplementary material. The raw sequencing reads used in this study were obtained from previously published datasets, as referenced in the Materials and methods (Data collection and sample preparation section). The processed data, including raw count tables and FASTA files of generated OTUs, are available on Figshare, https://doi.org/10.6084/m9.figshare.28847258.

## References

[ref1] Abdul Rahman N. Parks D. H. Willner D. L. Engelbrektson A. L. Goffredi S. K. Warnecke F.. (2015). A molecular survey of Australian and north American termite genera indicates that vertical inheritance is the primary force shaping termite gut microbiomes. Microbiome 3:5. doi: 10.1186/s40168-015-0067-8, PMID: 25830022 PMC4379614

[ref2] Abellan-Schneyder I. Matchado M. S. Reitmeier S. Sommer A. Sewald Z. Baumbach J.. (2021). Primer, pipelines, parameters: issues in 16S rRNA gene sequencing. mSphere 6:10.1128/msphere.01202-20. doi: 10.1128/msphere.01202-20 PMC8544895 33627512

[ref3] Aghnatios R. Drancourt M. (2016). Gemmata species: Planctomycetes of medical interest. Future Microbiol. 11, 659–667. doi: 10.2217/fmb-2015-0001, PMID: 27158864

[ref4] Arora J. Kinjo Y. Šobotník J. Buček A. Clitheroe C. Stiblik P.. (2022). The functional evolution of termite gut microbiota. Microbiome 10:78. doi: 10.1186/s40168-022-01258-3, PMID: 35624491 PMC9137090

[ref5] Bajpai P. (2020). “Chapter 7- advantages and disadvantages of biomass utilization” in Biomass to energy conversion technologies. ed. Bajpai P. (Elsevier), 169–173.

[ref6] Barberán A. Ramirez K. S. Leff J. W. Bradford M. A. Wall D. H. Fierer N. (2014). Why are some microbes more ubiquitous than others? Predicting the habitat breadth of soil bacteria. Ecol. Lett. 17, 794–802. doi: 10.1111/ele.12282, PMID: 24751288

[ref7] Bondoso J. Godoy-Vitorino F. Balagué V. Gasol J. M. Harder J. Lage O. M. (2017). Epiphytic Planctomycetes communities associated with three main groups of macroalgae. FEMS Microbiol. Ecol. 93:fiw255. doi: 10.1093/femsec/fiw255, PMID: 28087803 PMC5975077

[ref8] Bonechi C. Consumi M. Donati A. Leone G. Magnani A. Tamasi G.. (2017). “Biomass” in Bioenergy Systems for the Future. Eds, F. Dalena, A. Basile, C. Rossi (Woodhead Publishing), 3–42.

[ref9] Briliūtė J. Urbanowicz P. A. Luis A. S. Baslé A. Paterson N. Rebello O.. (2019). Complex N-glycan breakdown by gut Bacteroides involves an extensive enzymatic apparatus encoded by multiple co-regulated genetic loci. Nat. Microbiol. 4, 1571–1581. doi: 10.1038/s41564-019-0466-x, PMID: 31160824 PMC7617214

[ref10] Brune A. (2014). Symbiotic digestion of lignocellulose in termite guts. Nat. Rev. Microbiol. 12, 168–180. doi: 10.1038/nrmicro3182, PMID: 24487819

[ref11] Buckley D. H. Huangyutitham V. Nelson T. A. Rumberger A. Thies J. E. (2006). Diversity of Planctomycetes in soil in relation to soil history and environmental heterogeneity. AEM 72, 4522–4531. doi: 10.1128/AEM.00149-06, PMID: 16820439 PMC1489350

[ref12] Cabello-Yeves P. J. Callieri C. Picazo A. Mehrshad M. Haro-Moreno J. M. Roda-Garcia J. J.. (2021). The microbiome of the Black Sea water column analyzed by shotgun and genome centric metagenomics. Environ. Microbiome 16:5. doi: 10.1186/s40793-021-00374-1, PMID: 33902743 PMC8067304

[ref13] Callahan B. J. Wong J. Heiner C. Oh S. Theriot C. M. Gulati A. S.. (2019). High-throughput amplicon sequencing of the full-length 16S rRNA gene with single-nucleotide resolution. Nucleic Acids Res. 47:e103. doi: 10.1093/nar/gkz569, PMID: 31269198 PMC6765137

[ref14] Calusinska M. Goux X. Fossépré M. Muller E. E. L. Wilmes P. Delfosse P. (2018). A year of monitoring 20 mesophilic full-scale bioreactors reveals the existence of stable but different core microbiomes in bio-waste and wastewater anaerobic digestion systems. Biotechnol. Biofuels 11:196. doi: 10.1186/s13068-018-1195-8, PMID: 30038663 PMC6052691

[ref15] Calusinska M. Marynowska M. Bertucci M. Untereiner B. Klimek D. Goux X.. (2020). Integrative omics analysis of the termite gut system adaptation to Miscanthus diet identifies lignocellulose degradation enzymes. Commun. Biol. 3, 275–212. doi: 10.1038/s42003-020-1004-3, PMID: 32483294 PMC7264248

[ref16] Carter M. M. Olm M. R. Merrill B. D. Dahan D. Tripathi S. Spencer S. P.. (2023). Ultra-deep sequencing of Hadza hunter-gatherers recovers vanishing gut microbes. Cell 186, 3111–3124.e13. doi: 10.1016/j.cell.2023.05.046, PMID: 37348505 PMC10330870

[ref17] Cayrou C. Sambe B. Armougom F. Raoult D. Drancourt M. (2013). Molecular diversity of the Planctomycetes in the human gut microbiota in France and Senegal. APMIS 121, 1082–1090. doi: 10.1111/apm.12087, PMID: 23594317

[ref18] Chen H. (2014). Chemical composition and structure of natural lignocellulose, in: Biotechnology of lignocellulose. Dordrecht: Springer Netherlands, 25–71.

[ref19] Chen Q. Hu H. Yan Z. Li C. Nguyen B. T. Zheng Y.. (2021). Termite mounds reduce soil microbial diversity by filtering rare microbial taxa. Environ. Microbiol. 23, 2659–2668. doi: 10.1111/1462-2920.15507, PMID: 33817921

[ref20] Coronel M. D. M. Q. Devos D. P. Garcillán-Barcia M. P. (2024). Specificities and commonalities of the Planctomycetes plasmidome. Environ. Microbiol. 26:e16638. doi: 10.1101/2024.01.22.576637 38733104

[ref21] Dar M. A. Xie R. Jing L. Qing X. Ali S. Pandit R. S.. (2024). Elucidating the structure, and composition of bacterial symbionts in the gut regions of wood-feeding termite, *Coptotermes formosanus* and their functional profile towards lignocellulolytic systems. Front. Microbiol. 15:1395568. doi: 10.3389/fmicb.2024.1395568, PMID: 38846576 PMC11155305

[ref22] de Araujo J. E. Taketani R. G. Pylro V. S. Leite L. R. Silva M. C. P. E. Lemos L. N.. (2021). Genomic analysis reveals the potential for hydrocarbon degradation of Rhodopirellula sp. MGV isolated from a polluted Brazilian mangrove. Braz. J. Microbiol. 52, 1397–1404. doi: 10.1007/s42770-021-00483-6, PMID: 33852152 PMC8324706

[ref9001] Dedysh S. N. Beletsky A. V. Ivanova A. A. Kulichevskaya I. S. Suzina N. E. Philippov D. A.. (2021). Wide distribution of Phycisphaera-like planctomycetes from WD2101 soil group in peatlands and genome analysis of the first cultivated representative. Environ Microbiol. 23, 1510–1526. doi: 10.1111/1462-2920.15360 33325093

[ref9002] Dedysh S. N. Henke P. Ivanova A. A. Kulichevskaya I. S. Philippov D. A. Meier-Kolthoff J. P.. (2020). 100-year-old enigma solved: identification, genomic characterization and biogeography of the yet uncultured Planctomyces bekefii. Environ Microbiol. 22, 198–211. doi: 10.1111/1462-2920.14838 31637799

[ref24] Dedysh S. N. Ivanova A. A. (2019). Planctomycetes in boreal and subarctic wetlands: diversity patterns and potential ecological functions. FEMS Microbiol. Ecol. 95:fiy227. doi: 10.1093/femsec/fiy227, PMID: 30476049

[ref23] De Mandal S. Chatterjee R. Kumar N. S. (2017). Dominant bacterial phyla in caves and their predicted functional roles in C and N cycle. BMC Microbiol. 17:90. doi: 10.1186/s12866-017-1002-x, PMID: 28399822 PMC5387202

[ref25] Drula E. Garron M.-L. Dogan S. Lombard V. Henrissat B. Terrapon N. (2022). The carbohydrate-active enzyme database: functions and literature. Nucleic Acids Res. 50, D571–D577. doi: 10.1093/nar/gkab1045, PMID: 34850161 PMC8728194

[ref26] Edgar R. C. (2018). Updating the 97% identity threshold for 16S ribosomal RNA OTUs. Bioinformatics 34, 2371–2375. doi: 10.1093/bioinformatics/bty113, PMID: 29506021

[ref27] Eilmus S. Heil M. (2009). Bacterial Associates of Arboreal Ants and Their Putative Functions in an obligate ant-plant mutualism. AEM 75, 4324–4332. doi: 10.1128/AEM.00455-09, PMID: 19447959 PMC2704814

[ref28] Elshahed M. S. Youssef N. H. Luo Q. Najar F. Z. Roe B. A. Sisk T. M.. (2007). Phylogenetic and metabolic diversity of Planctomycetes from anaerobic, sulfide-and sulfur-rich Zodletone Spring. Oklahoma. Appl. Environ. Microbiol. 73, 4707–4716. doi: 10.1128/AEM.00591-07, PMID: 17545322 PMC1951033

[ref29] Farias M. E. Rasuk M. C. Gallagher K. L. Contreras M. Kurth D. Fernandez A. B.. (2017). Prokaryotic diversity and biogeochemical characteristics of benthic microbial ecosystems at La Brava, a hypersaline lake at Salar de Atacama, Chile. PLoS ONE 12:e0186867. doi: 10.1371/journal.pone.0186867, PMID: 29140980 PMC5687714

[ref30] Fernández-Gómez B. Richter M. Schüler M. Pinhassi J. Acinas S. G. González J. M.. (2013). Ecology of marine Bacteroidetes: a comparative genomics approach. ISME J. 7, 1026–1037. doi: 10.1038/ismej.2012.169, PMID: 23303374 PMC3635232

[ref31] Figueroa D. Capo E. Lindh M. V. Rowe O. F. Paczkowska J. Pinhassi J.. (2021). Terrestrial dissolved organic matter inflow drives temporal dynamics of the bacterial community of a subarctic estuary (northern Baltic Sea). Environ. Microbiol. 23, 4200–4213. doi: 10.1111/1462-2920.15597, PMID: 33998121

[ref32] Flores C. Catita J. A. M. Lage O. M. (2014). Assessment of planctomycetes cell viability after pollutants exposure. Antonie Van Leeuwenhoek 106, 399–411. doi: 10.1007/s10482-014-0206-4, PMID: 24903954

[ref33] Gharechahi J. Sarikhan S. Han J.-L. Ding X.-Z. Salekdeh G. H. (2022). Functional and phylogenetic analyses of camel rumen microbiota associated with different lignocellulosic substrates. npj Biofilms Microbiomes 8:46. doi: 10.1038/s41522-022-00309-9, PMID: 35676509 PMC9177762

[ref34] Godinho O. Botelho R. Albuquerque L. Wiegand S. Kallscheuer N. da Costa M. S.. (2021). Bremerella alba sp. nov., a novel planctomycete isolated from the surface of the macroalga *Fucus spiralis*. Syst. Appl. Microbiol. 44:126189. doi: 10.1016/j.syapm.2021.126189, PMID: 33852992

[ref35] González Plaza J. J. Hradecký J. (2023). The tropical cookbook: termite diet and phylogenetics—over geographical origin—drive the microbiome and functional genetic structure of nests. Front. Microbiol. 14:1089525. doi: 10.3389/fmicb.2023.1089525, PMID: 36998409 PMC10043212

[ref36] Goux X. Liu T. Westerholm M. Calusinska M. (2023). “Microbial degradation of lignocellulose in natural and engineered systems – from the smallest to the biggest bioreactor” in Microbial fermentations in nature and as designed processes (Hoboken, United States: John Wiley & Sons, Ltd), 167–205.

[ref37] Hagey J. V. Bhatnagar S. Heguy J. M. Karle B. M. Price P. L. Meyer D.. (2019). Fecal microbial communities in a large representative cohort of California dairy cows. Front. Microbiol. 10:1093. doi: 10.3389/fmicb.2019.01093, PMID: 31156599 PMC6532609

[ref38] Hervé V. Liu P. Dietrich C. Sillam-Dussès D. Stiblik P. Šobotník J.. (2020). Phylogenomic analysis of 589 metagenome-assembled genomes encompassing all major prokaryotic lineages from the gut of higher termites. PeerJ 8:e8614. doi: 10.7717/peerj.8614, PMID: 32095380 PMC7024585

[ref39] Howe K. L. Seitz K. W. Campbell L. G. Baker B. J. Thrash J. C. Rabalais N. N.. (2023). Metagenomics and metatranscriptomics reveal broadly distributed, active, novel methanotrophs in the Gulf of Mexico hypoxic zone and in the marine water column. FEMS Microbiol. Ecol. 99:fiac153. doi: 10.1093/femsec/fiac153, PMID: 36520069 PMC9874027

[ref40] Hu X. Gu H. Liu J. Wei D. Zhu P. Cui X.. (2022). Metagenomics reveals divergent functional profiles of soil carbon and nitrogen cycling under long-term addition of chemical and organic fertilizers in the black soil region. Geoderma 418:115846. doi: 10.1016/j.geoderma.2022.115846

[ref41] Ivanova A. A. Kulichevskaya I. S. Merkel A. Y. Toshchakov S. V. Dedysh S. N. (2016). High diversity of Planctomycetes in soils of two lichen-dominated sub-Arctic ecosystems of northwestern Siberia. Front. Microbiol. 7:2065. doi: 10.3389/fmicb.2016.02065, PMID: 28066382 PMC5177623

[ref42] Ivanova A. A. Naumoff D. G. Kulichevskaya I. S. Rakitin A. L. Mardanov A. V. Ravin N. V.. (2024). Planctomycetes of the genus Singulisphaera possess Chitinolytic capabilities. Microorganisms 12:1266. doi: 10.3390/microorganisms12071266, PMID: 39065035 PMC11279305

[ref43] Ivanova A. A. Naumoff D. G. Miroshnikov K. K. Liesack W. Dedysh S. N. (2017). Comparative genomics of four Isosphaeraceae Planctomycetes: a common Pool of plasmids and glycoside hydrolase genes shared by Paludisphaera borealis PX4T, *Isosphaera pallida* IS1BT, *Singulisphaera acidiphila* DSM 18658T, and strain SH-PL62. Front. Microbiol. 8:412. doi: 10.3389/fmicb.2017.00412, PMID: 28360896 PMC5352709

[ref44] Ivanova A. A. Wegner C.-E. Kim Y. Liesack W. Dedysh S. N. (2018). Metatranscriptomics reveals the hydrolytic potential of peat-inhabiting Planctomycetes. Antonie Van Leeuwenhoek 111, 801–809. doi: 10.1007/s10482-017-0973-9, PMID: 29134393

[ref45] Jiang B. Zeng Q. Liu J. Hou Y. Xu J. Li H.. (2020). Enhanced treatment performance of phenol wastewater and membrane antifouling by biochar-assisted EMBR. Bioresour. Technol. 306:123147. doi: 10.1016/j.biortech.2020.123147, PMID: 32171174

[ref46] Johnson J. S. Spakowicz D. J. Hong B.-Y. Petersen L. M. Demkowicz P. Chen L.. (2019). Evaluation of 16S rRNA gene sequencing for species and strain-level microbiome analysis. Nat. Commun. 10:5029. doi: 10.1038/s41467-019-13036-1, PMID: 31695033 PMC6834636

[ref47] Kaboré O. D. Godreuil S. Drancourt M. (2020). Planctomycetes as host-associated Bacteria: a perspective that holds promise for their future isolations, by mimicking their native environmental niches in clinical microbiology laboratories. Front. Cell. Infect. Microbiol. 10:519301. doi: 10.3389/fcimb.2020.519301, PMID: 33330115 PMC7734314

[ref48] Kallscheuer N. Rast P. Jogler M. Wiegand S. Kohn T. Boedeker C.. (2021). Analysis of bacterial communities in a municipal duck pond during a phytoplankton bloom and isolation of Anatilimnocola aggregata gen. Nov., sp. nov., Lacipirellula limnantheis sp. nov. and Urbifossiella limnaea gen. Nov., sp. nov. belonging to the phylum Planctomycetes. Environ. Microbiol. 23, 1379–1396. doi: 10.1111/1462-2920.15341, PMID: 33331109

[ref9003] Kallscheuer N. Wiegand S. Boedeker C. Peeters S. H. Jogler M. Heuer A.. (2020b). Caulifigura coniformis gen. nov., sp. nov., a novel member of the family Planctomycetaceae isolated from a red biofilm sampled in a hydrothermal area. Antonie Van Leeuwenhoek. 113, 1927–1937. doi: 10.1007/s10482-020-01439-w 32583190 PMC7717036

[ref49] Kallscheuer N. Wiegand S. Boedeker C. Peeters S. H. Jogler M. Rast P.. (2020a). *Aureliella helgolandensis* gen. Nov., sp. nov., a novel Planctomycete isolated from a jellyfish at the shore of the island Helgoland. Antonie Van Leeuwenhoek 113, 1839–1849. doi: 10.1007/s10482-020-01403-8 32219667 PMC7716919

[ref50] Kappelmann L. Krüger K. Hehemann J.-H. Harder J. Markert S. Unfried F.. (2019). Polysaccharide utilization loci of North Sea Flavobacteriia as basis for using SusC/D-protein expression for predicting major phytoplankton glycans. ISME J. 13, 76–91. doi: 10.1038/s41396-018-0242-6, PMID: 30111868 PMC6298971

[ref9004] Kaushik R. Sharma M. Gaurav K. Jagadeeshwari U. Shabbir A. Sasikala C.. (2020). Paludisphaera soli sp. nov., a new member of the family Isosphaeraceae isolated from high altitude soil in the Western Himalaya. Antonie Van Leeuwenhoek. 113, 1663–1674. doi: 10.1007/s10482-020-01471-w 32936355

[ref51] Kembel S. W. Cowan P. D. Helmus M. R. Cornwell W. K. Morlon H. Ackerly D. D.. (2010). Picante: R tools for integrating phylogenies and ecology. Bioinformatics 26, 1463–1464. doi: 10.1093/bioinformatics/btq166, PMID: 20395285

[ref52] Kim J. W. Brawley S. H. Prochnik S. Chovatia M. Grimwood J. Jenkins J.. (2016). Genome analysis of Planctomycetes inhabiting blades of the red alga *Porphyra umbilicalis*. PLoS One 11:e0151883. doi: 10.1371/journal.pone.0151883, PMID: 27015628 PMC4807772

[ref53] Kim M. Kim J. Kuehn L. A. Bono J. L. Berry E. D. Kalchayanand N.. (2014). Investigation of bacterial diversity in the feces of cattle fed different diets1. J. Anim. Sci. 92, 683–694. doi: 10.2527/jas.2013-6841 24352967

[ref54] Klimek D. Herold M. Calusinska M. (2024). Comparative genomic analysis of Planctomycetota potential for polysaccharide degradation identifies biotechnologically relevant microbes. BMC Genomics 25:523. doi: 10.1186/s12864-024-10413-z, PMID: 38802741 PMC11131199

[ref55] Klimek D. Herold M. Vitorino I. R. Dedova Z. Lemaigre S. Roussel J.. (2025). Insights into the phylogenetic and metabolic diversity of Planctomycetota in anaerobic digesters and the isolation of novel Thermoguttaceae species. FEMS Microbiol. Ecol. 101:fiaf025. doi: 10.1093/femsec/fiaf025, PMID: 40097306 PMC11929135

[ref56] Köhler T. Stingl U. Meuser K. Brune A. (2008). Novel lineages of Planctomycetes densely colonize the alkaline gut of soil-feeding termites (Cubitermes spp.). Environ. Microbiol. 10, 1260–1270. doi: 10.1111/j.1462-2920.2007.01540.x, PMID: 18279348

[ref57] Kulichevskaya I. S. Detkova E. N. Bodelier P. L. E. Rijpstra W. I. C. Sinninghe Damsté J. S. Dedysh S. N. (2012). *Singulisphaera rosea* sp. nov., a planctomycete from acidic Sphagnum peat, and emended description of the genus Singulisphaera. Int. J. Syst. Evol. Microbiol. 62, 118–123. doi: 10.1099/ijs.0.025924-0, PMID: 21335501

[ref9005] Kulichevskaya I. S. Ivanova A. O. Belova S. E. Baulina O. I. Bodelier P. L. E. Rijpstra W. I. C.. (2007). Schlesneria paludicola gen. nov., sp. nov., the first acidophilic member of the order Planctomycetales, from Sphagnum-dominated boreal wetlands. Int J Syst Evol Microbiol. 57, 2680–2687. doi: 10.1099/ijs.0.65157-0 17978240

[ref9006] Kulichevskaya I. S. Ivanova A. A. Naumoff D. G. Beletsky A. V. Rijpstra W. I. C. Sinninghe Damsté J. S.. (2020). Frigoriglobus tundricola gen. nov., sp. nov., a psychrotolerant cellulolytic planctomycete of the family Gemmataceae from a littoral tundra wetland. Syst Appl Microbiol. 43:126129. doi: 10.1016/j.syapm.2020.126129 32847778 PMC7534041

[ref58] Lee W.-J. Hase K. (2014). Gut microbiota–generated metabolites in animal health and disease. Nat. Chem. Biol. 10, 416–424. doi: 10.1038/nchembio.1535, PMID: 24838170

[ref59] Li D. Chen J. Zhang X. Shi W. Li J. (2022). Structural and functional characteristics of soil microbial communities in response to different ecological risk levels of heavy metals. Front. Microbiol. 13:1072389. doi: 10.3389/fmicb.2022.1072389, PMID: 36569064 PMC9772559

[ref60] Li M. Jain S. Dick G. J. (2016). Genomic and transcriptomic resolution of organic matter utilization among Deep-Sea Bacteria in Guaymas Basin hydrothermal plumes. Front. Microbiol. 7:1125. doi: 10.3389/fmicb.2016.01125, PMID: 27512389 PMC4962555

[ref61] Lillington S. P. Leggieri P. A. Heom K. A. O’Malley M. A. (2020). Nature’s recyclers: anaerobic microbial communities drive crude biomass deconstruction. Curr. Opin. Biotechnol. 62, 38–47. doi: 10.1016/j.copbio.2019.08.015, PMID: 31593910

[ref62] Liu J. Bai Y. Liu F. Kohn R. A. Tadesse D. A. Sarria S.. (2022). Rumen microbial predictors for short-chain fatty acid levels and the grass-fed regimen in Angus cattle. Animals 12:2995. doi: 10.3390/ani12212995, PMID: 36359118 PMC9656057

[ref63] Liu T. Schnürer A. Björkmalm J. Willquist K. Kreuger E. (2020). Diversity and abundance of microbial communities in UASB reactors during methane production from hydrolyzed wheat straw and Lucerne. Microorganisms 8:1394. doi: 10.3390/microorganisms8091394, PMID: 32932830 PMC7565072

[ref64] López-Mondéjar R. Tláskal V. da Rocha U. N. Baldrian P. (2022). Global distribution of carbohydrate utilization potential in the prokaryotic tree of life. mSystems 7:e0082922. doi: 10.1128/msystems.00829-22, PMID: 36413015 PMC9765126

[ref65] Lozupone C. Knight R. (2005). UniFrac: a new phylogenetic method for comparing microbial communities. Appl. Environ. Microbiol. 71, 8228–8235. doi: 10.1128/AEM.71.12.8228-8235.2005, PMID: 16332807 PMC1317376

[ref66] Mach N. Midoux C. Leclercq S. Pennarun S. Le Moyec L. Rué O.. (2022). Mining the equine gut metagenome: poorly-characterized taxa associated with cardiovascular fitness in endurance athletes. Commun. Biol. 5, 1–15. doi: 10.1038/s42003-022-03977-7, PMID: 36192523 PMC9529974

[ref67] Malard L. A. Pearce D. A. (2018). Microbial diversity and biogeography in Arctic soils. Environ. Microbiol. Rep. 10, 611–625. doi: 10.1111/1758-2229.12680, PMID: 30028082

[ref68] Mao S. Zhang R. Wang D. Zhu W. (2012). The diversity of the fecal bacterial community and its relationship with the concentration of volatile fatty acids in the feces during subacute rumen acidosis in dairy cows. BMC Vet. Res. 8:237. doi: 10.1186/1746-6148-8-237, PMID: 23217205 PMC3582618

[ref69] Martin W. F. Sousa F. L. (2016). Early microbial evolution: the age of anaerobes. Cold Spring Harb. Perspect. Biol. 8:a018127. doi: 10.1101/cshperspect.a018127, PMID: 26684184 PMC4743081

[ref70] Marynowska M. Goux X. Sillam-Dussès D. Rouland-Lefèvre C. Halder R. Wilmes P.. (2020). Compositional and functional characterisation of biomass-degrading microbial communities in guts of plant fibre-and soil-feeding higher termites. Microbiome 8:96. doi: 10.1186/s40168-020-00872-3, PMID: 32576253 PMC7313118

[ref71] Marynowska M. Sillam-Dussès D. Untereiner B. Klimek D. Goux X. Gawron P.. (2023). A holobiont approach towards polysaccharide degradation by the highly compartmentalised gut system of the soil-feeding higher termite Labiotermes labralis. BMC Genomics 24:115. doi: 10.1186/s12864-023-09224-5, PMID: 36922761 PMC10018900

[ref72] Medina-Silva R. Oliveira R. R. Trindade F. J. Borges L. G. A. Lopes Simão T. L. Augustin A. H.. (2018). Microbiota associated with tubes of *Escarpia* sp. from cold seeps in the southwestern Atlantic Ocean constitutes a community distinct from that of surrounding marine sediment and water. Antonie Van Leeuwenhoek 111, 533–550. doi: 10.1007/s10482-017-0975-7, PMID: 29110156

[ref73] Mikaelyan A. Meuser K. Brune A. (2017). Microenvironmental heterogeneity of gut compartments drives bacterial community structure in wood-and humus-feeding higher termites. FEMS Microbiol. Ecol. 93:fiw210. doi: 10.1093/femsec/fiw210, PMID: 27798065

[ref74] Mote R. S. Hill N. S. Skarlupka J. H. Turner Z. B. Sanders Z. P. Jones D. P.. (2019). Response of beef cattle fecal microbiota to grazing on toxic tall fescue. Appl. Environ. Microbiol. 85, e00032–e00019. doi: 10.1128/AEM.00032-19, PMID: 31126949 PMC6643230

[ref75] Nguyen L. N. Nguyen A. Q. Nghiem L. D. (2019). “Microbial Community in Anaerobic Digestion System: progression in microbial ecology” in Water and wastewater treatment technologies. eds. Bui X.-T. Chiemchaisri C. Fujioka T. Varjani S. (Singapore: Springer), 331–355.

[ref76] Nunes da Rocha U. Van Overbeek L. Van Elsas J. D. (2009). Exploration of hitherto-uncultured bacteria from the rhizosphere. FEMS Microbiol. Ecol. 69, 313–328. doi: 10.1111/j.1574-6941.2009.00702.x, PMID: 19508698

[ref77] Ohkuma M. Brune A. (2011). “Diversity, structure, and evolution of the termite gut microbial community” in Biology of termites: A modern synthesis. eds. Bignell D. E. Roisin Y. Lo N. (Dordrecht: Springer), 413–438.

[ref78] Oksanen J. Simpson G. L. Blanchet F. G. Kindt R. Legendre P. Minchin P. R.. (2001). Vegan: Community ecology package. doi: 10.32614/CRAN.package.vegan

[ref79] Partanen P. Hultman J. Paulin L. Auvinen P. Romantschuk M. (2010). Bacterial diversity at different stages of the composting process. BMC Microbiol. 10:94. doi: 10.1186/1471-2180-10-94, PMID: 20350306 PMC2907838

[ref80] Quast C. Pruesse E. Yilmaz P. Gerken J. Schweer T. Yarza P.. (2013). The SILVA ribosomal RNA gene database project: improved data processing and web-based tools. Nucleic Acids Res. 41, D590–D596. doi: 10.1093/nar/gks1219, PMID: 23193283 PMC3531112

[ref81] Ravinath R. Das A. J. Usha T. Ramesh N. Middha S. K. (2022). Targeted metagenome sequencing reveals the abundance of Planctomycetes and Bacteroidetes in the rhizosphere of pomegranate. Arch. Microbiol. 204:481. doi: 10.1007/s00203-022-03100-8, PMID: 35834016

[ref82] Reichart N. J. Bowers R. M. Woyke T. Hatzenpichler R. (2021). High potential for biomass-degrading enzymes revealed by hot Spring metagenomics. Front. Microbiol. 12:668238. doi: 10.3389/fmicb.2021.668238, PMID: 33968004 PMC8098120

[ref83] Richards C. Otani S. Mikaelyan A. Poulsen M. (2017). *Pycnoscelus surinamensis* cockroach gut microbiota respond consistently to a fungal diet without mirroring those of fungus-farming termites. PLoS One 12:e0185745. doi: 10.1371/journal.pone.0185745, PMID: 28973021 PMC5626473

[ref84] Sabree Z. L. Moran N. A. (2014). Host-specific assemblages typify gut microbial communities of related insect species. Springerplus 3:138. doi: 10.1186/2193-1801-3-138, PMID: 24741474 PMC3979980

[ref85] Salgado J. F. M. Hervé V. Vera M. A. G. Tokuda G. Brune A. (2024). Unveiling lignocellulolytic potential: a genomic exploration of bacterial lineages within the termite gut. Microbiome 12:201. doi: 10.1186/s40168-024-01917-7, PMID: 39407345 PMC11481507

[ref86] Schloss P. D. (2021). Amplicon sequence variants artificially Split bacterial genomes into separate clusters. mSphere 6:e0019121. doi: 10.1128/msphere.00191-21 34287003 PMC8386465

[ref87] Schloss P. D. Westcott S. L. Ryabin T. Hall J. R. Hartmann M. Hollister E. B.. (2009). Introducing mothur: open-source, platform-independent, community-supported software for describing and comparing microbial communities. Appl. Environ. Microbiol. 75, 7537–7541. doi: 10.1128/AEM.01541-09, PMID: 19801464 PMC2786419

[ref88] Sethupathy S. Morales G. M. Li Y. Wang Y. Jiang J. Sun J.. (2021). Harnessing microbial wealth for lignocellulose biomass valorization through secretomics: a review. Biotechnol. Biofuels 14:154. doi: 10.1186/s13068-021-02006-9, PMID: 34225772 PMC8256616

[ref89] Sharma P. Sharma S. Maurya R. K. De T. D. Thomas T. Lata S.. (2014). Salivary glands harbor more diverse microbial communities than gut in *Anopheles culicifacies*. Parasit. Vectors 7:235. doi: 10.1186/1756-3305-7-235, PMID: 24886293 PMC4062515

[ref90] Shen J. Smith A. C. Barnett M. J. Morgan A. Wynn P. M. (2022). Distinct microbial communities in the soils, waters, and speleothems of a Hyperalkaline cave system. Journal of geophysical research. Biogeosciences 127:e2022JG006866. doi: 10.1029/2022JG006866

[ref91] Singh N. Singh V. Rai S. N. Vamanu E. Singh M. P. (2022). Metagenomic analysis of garden soil-derived microbial consortia and unveiling their metabolic potential in mitigating toxic hexavalent chromium. Life 12:2094. doi: 10.3390/life12122094, PMID: 36556458 PMC9781466

[ref92] Slobodkina G. B. Kovaleva O. L. Miroshnichenko M. L. Slobodkin A. I. Kolganova T. V. Novikov A. A.. (2015). *Thermogutta terrifontis* gen. Nov., sp. nov. and Thermogutta hypogea sp. nov., thermophilic anaerobic representatives of the phylum Planctomycetes. Int. J. Syst. Evol. Microbiol. 65, 760–765. doi: 10.1099/ijs.0.000009, PMID: 25479950

[ref93] Slobodkina G. B. Panteleeva A. N. Beskorovaynaya D. A. Bonch-Osmolovskaya E. A. Slobodkin A. I. (2016). *Thermostilla marina* gen. Nov., sp. nov., a thermophilic, facultatively anaerobic planctomycete isolated from a shallow submarine hydrothermal vent. Int. J. Syst. Evol. Microbiol. 66, 633–638. doi: 10.1099/ijsem.0.000767, PMID: 26559645

[ref94] Song H. Peng L. Li Z. Deng X. Shao J. Gu J. (2019). Metal distribution and biological diversity of crusts in paddy fields polluted with different levels of cadmium. Ecotoxicol. Environ. Saf. 184:109620. doi: 10.1016/j.ecoenv.2019.109620, PMID: 31493587

[ref95] Spring S. Brinkmann N. Murrja M. Spröer C. Reitner J. Klenk H.-P. (2015). High diversity of Culturable prokaryotes in a lithifying hypersaline microbial mat. Geomicrobiol J. 32, 332–346. doi: 10.1080/01490451.2014.913095

[ref96] Stewart R. D. Auffret M. D. Warr A. Walker A. W. Roehe R. Watson M. (2019). Compendium of 4,941 rumen metagenome-assembled genomes for rumen microbiome biology and enzyme discovery. Nat. Biotechnol. 37, 953–961. doi: 10.1038/s41587-019-0202-3, PMID: 31375809 PMC6785717

[ref97] Storesund J. E. Lanzèn A. Nordmann E.-L. Armo H. R. Lage O. M. Øvreås L. (2020). Planctomycetes as a vital constituent of the microbial communities inhabiting different layers of the meromictic Lake Sælenvannet (Norway). Microorganisms 8:1150. doi: 10.3390/microorganisms8081150, PMID: 32751313 PMC7464441

[ref98] Sun C.-C. Zhao W.-J. Yue W.-Z. Cheng H. Sun F.-L. Wang Y.-T.. (2023). Polymeric carbohydrates utilization separates microbiomes into niches: Insights into the diversity of microbial carbohydrate-active enzymes in the inner shelf of the Pearl River Estuary, China. Front. Microbiol. 14:1180321. doi: 10.3389/fmicb.2023.1180321 37425997 PMC10322874

[ref99] Suto R. Ishimoto C. Chikyu M. Aihara Y. Matsumoto T. Uenishi H.. (2017). Anammox biofilm in activated sludge swine wastewater treatment plants. Chemosphere 167, 300–307. doi: 10.1016/j.chemosphere.2016.09.121, PMID: 27728889

[ref100] Tran N. T. Zhang J. Xiong F. Wang G.-T. Li W.-X. Wu S.-G. (2018). Altered gut microbiota associated with intestinal disease in grass carp (*Ctenopharyngodon idellus*). World J. Microbiol. Biotechnol. 34:71. doi: 10.1007/s11274-018-2447-2, PMID: 29777414

[ref101] Vanwonterghem I. Jensen P. D. Rabaey K. Tyson G. W. (2016). Genome-centric resolution of microbial diversity, metabolism and interactions in anaerobic digestion. Environ. Microbiol. 18, 3144–3158. doi: 10.1111/1462-2920.13382, PMID: 27317862

[ref102] Vargas-Albores F. Porchas-Cornejo M. A. Martínez-Porchas M. Villalpando-Canchola E. Gollas-Galván T. Martínez-Córdova L. R. (2017). Bacterial biota of shrimp intestine is significantly modified by the use of a probiotic mixture: a high throughput sequencing approach. Helgol. Mar. Res. 71:5. doi: 10.1186/s10152-017-0485-z

[ref103] Vipindas P. V. Krishnan K. P. Rehitha T. V. Jabir T. Dinesh S. L. (2020). Diversity of sediment associated Planctomycetes and its related phyla with special reference to anammox bacterial community in a high Arctic fjord. World J. Microbiol. Biotechnol. 36:107. doi: 10.1007/s11274-020-02886-3, PMID: 32638161

[ref104] Vitorino I. R. Lage O. M. (2022). The Planctomycetia: an overview of the currently largest class within the phylum Planctomycetes. Antonie Van Leeuwenhoek 115, 169–201. doi: 10.1007/s10482-021-01699-0, PMID: 35037113

[ref105] Vollmers J. Frentrup M. Rast P. Jogler C. Kaster A.-K. (2017). Untangling genomes of novel Planctomycetal and Verrucomicrobial species from Monterey Bay kelp Forest metagenomes by refined binning. Front. Microbiol. 8:472. doi: 10.3389/fmicb.2017.00472, PMID: 28424662 PMC5372823

[ref106] Walters K. E. Martiny J. B. H. (2020). Alpha-, beta-, and gamma-diversity of bacteria varies across habitats. PLoS One 15:e0233872. doi: 10.1371/journal.pone.0233872, PMID: 32966309 PMC7510982

[ref107] Wang X. Cao A. Zhao G. Zhou C. Xu R. (2017). Microbial community structure and diversity in a municipal solid waste landfill. Waste Manag. 66, 79–87. doi: 10.1016/j.wasman.2017.04.023, PMID: 28442259

[ref108] Wang C. Dong D. Wang H. Müller K. Qin Y. Wang H.. (2016). Metagenomic analysis of microbial consortia enriched from compost: new insights into the role of Actinobacteria in lignocellulose decomposition. Biotechnol. Biofuels 9:22. doi: 10.1186/s13068-016-0440-2, PMID: 26834834 PMC4731972

[ref109] Wang Y. Fu Y. He Y. Kulyar M. F.-A. Iqbal M. Li K.. (2021). Longitudinal characterization of the gut bacterial and fungal communities in yaks. J. Fungi 7:559. doi: 10.3390/jof7070559, PMID: 34356938 PMC8304987

[ref9007] Wiegand S. Jogler M. Boedeker C. Heuer A. Peeters S. H. Kallscheuer N.. (2020). Updates to the recently introduced family Lacipirellulaceae in the phylum Planctomycetes: isolation of strains belonging to the novel genera Aeoliella, Botrimarina, Pirellulimonas and Pseudobythopirellula and the novel species Bythopirellula polymerisocia and Posidoniimonas corsicana. Antonie Van Leeuwenhoek. 113, 1979–1997. doi: 10.1007/s10482-020-01486-3 33151460 PMC7717034

[ref110] Weimer P. J. (2022). Degradation of cellulose and hemicellulose by ruminal microorganisms. Microorganisms 10:2345. doi: 10.3390/microorganisms10122345 36557598 PMC9785684

[ref111] Xiao H. Yu H. Wang J. He L. Wang Z. Fu Y.. (2025). Reforestation practices have varied the resilience of nosZ-type denitrifier communities: a 40-year soil chronosequence study. Appl. Soil Ecol. 206:105877. doi: 10.1016/j.apsoil.2025.105877

[ref112] Yang G. L. Hou S. G. Le Baoge R. Li Z. G. Xu H. Liu Y. P.. (2016). Differences in bacterial diversity and communities between glacial snow and glacial soil on the Chongce ice cap, West Kunlun Mountains. Sci. Rep. 6:36548. doi: 10.1038/srep36548, PMID: 27811967 PMC5109912

[ref113] Yildirim S. Yeoman C. J. Sipos M. Torralba M. Wilson B. A. Goldberg T. L.. (2010). Characterization of the fecal microbiome from non-human wild Primates reveals species specific microbial communities. PLoS One 5:e13963. doi: 10.1371/journal.pone.0013963, PMID: 21103066 PMC2980488

[ref114] Youssef N. Steidley B. L. Elshahed M. S. (2012). Novel high-rank phylogenetic lineages within a sulfur Spring (Zodletone Spring, Oklahoma), revealed using a combined pyrosequencing-sanger approach. Appl. Environ. Microbiol. 78, 2677–2688. doi: 10.1128/AEM.00002-12, PMID: 22307312 PMC3318811

[ref115] Žifčáková L. Větrovský T. Lombard V. Henrissat B. Howe A. Baldrian P. (2017). Feed in summer, rest in winter: microbial carbon utilization in forest topsoil. Microbiome 5:122. doi: 10.1186/s40168-017-0340-0, PMID: 28923122 PMC5604414

